# Supplementary figures and images for: Inhibition of Influenza Virus Replication by Oseltamivir Derivatives
Source: Pathogens. 2022 Feb 11;11(2):237. doi: 10.3390/pathogens11020237 (PMC8879189; doi:10.3390/pathogens11020237)

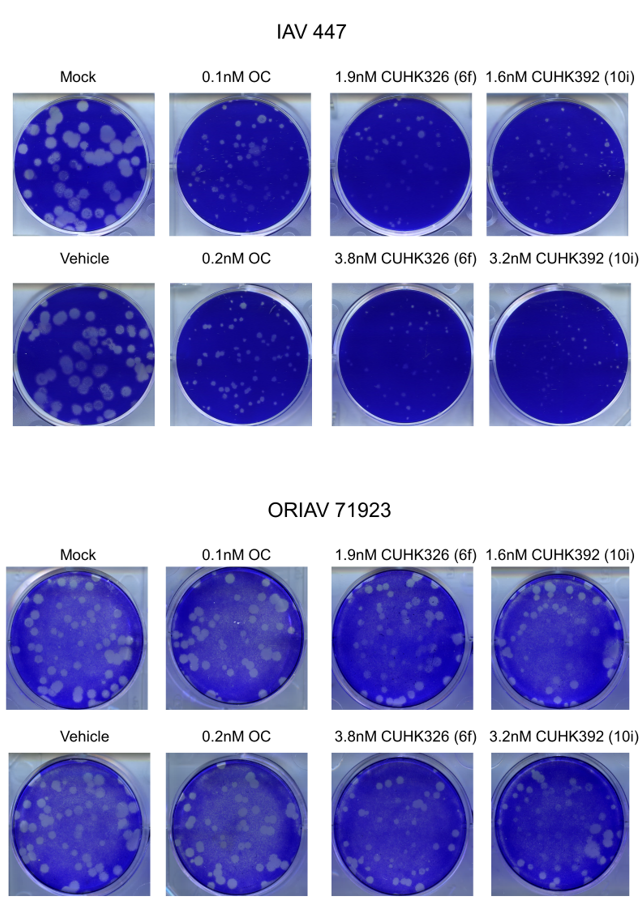

Supplement: Supplementary file 1 [file pathogens-11-00237-s001.zip › Figure S2.jpg]
